# Meeting Reports

**DOI:** 10.1155/S1463924680000132

**Published:** 1980


### Meeting Reports

### The 9th Technicon international symposium

The 9th Technicon International Symposium was held from the 14 - 16 November 1979 at the Maison de la Chimie in Paris. During the three days of the symposium speakers covered a wide range of topics related to automated analysis in clinical and industrial chemistry. A number of discussion sessions were also arranged. Additionally, working demonstrations of the complete range of Technicon products, including several new systems, were available for delegates to inspect and to discuss with attendant members of Technicon staff.

As is usual practice at these Technicon symposia, the programme was organised into parallel sessions – up to four in number, two with a clinical bias and two with an industrial bias. Whilst this approach may be satisfactory for people with fairly narrow interests, (in fairness to Technicon, probably the majority), it can be very irritating to participants with wider interests in automation. Almost invariably the more interesting papers in each session appeared to be arranged to take place simultaneously.

A representative sampling of the papers that were presented yielded rather disappointing results. Anyone who attended last year's Technicon Congress in London would have experienced a feeling of déjà vu. Indeed, a comparison with with the heyday of Technicon Symposia between 1965 and 1972, would not reflect favourably on present day standards. An unacceptably large proportion of speakers had very little that was new to offer. Many gave pedestrian accounts of how Technicon systems had been installed and used, with occasional tabulations of figures collected. To spend 20 minutes, as some speakers did, just to get across the message that an AutoAnalyzer can improve the quality of analytical data and save you money may gratify Technicon, but it bored at least one member of the audience. In the series of symposia that were held between 1965 and 1972 one heard a fair proportion of papers describing new applications of AutoAnalyzers, frequently with the use of modified modules and specially constructed components. Perhaps people can no longer afford the time to do such innovatory work. A sad thought, but hopefully wrong?

The equipment exhibition was well laid out with the clinical exhibits enjoying the lion's share of the space. The most interesting new exhibits in the Industrial section were a system to determine the original gravity of beer, the InfraAlyzer 300 and the AA-II-C. The original gravity instrument uses an Anton Paar DMA-55 Densimeter to measure the density of the sample and a Technicon thermometric analyser to measure the alcohol content, an associated calculator performs all the necessary calculations. The InfraAlyzer 300, which is a simplified and lower cost version of the InfraAlyzer 400, has been designed specifically for the analysis of grain and oil seeds. The AA-II-C is a single channel system with a microprocessor to control the operation of the sampler, the pump and a reagent-selection valve, as well as to perform all necessary calculations. The operating programs are entered via a teletype keyboard and the processor performs a wide range of calculations, drift corrections etc. as well as controlling the operation of the system. The most interesting innovation in the Clinical section was the Quantachem, Technicon's latest attempt to break into the discrete sample analyser market. It is an elegant as well as impressive instrument and it is obvious that considerable effort has been invested in keeping it mechanically simple and therefore, hopefully, reliable.

These Technicon congresses are large scale affairs attended by up to 2000 people who fortunately do not all arrive at once. Technicon (France) are to be congratulated on the running of the meeting, particularly as they had to overcome accommodation problems that did not arise during last year's symposium which was held at the Wembley Conference Centre, a new building designed specifically to cater for modern large scale conferences. The space available at the Maison de la Chimie is severely restricted but registration proceeded smoothly, nevertheless. However, for foreign delegates in particular, the Maison de la Chimie has an overriding advantage over Wembley as it is situated in the centre of Paris whereas Wembley is probably one of the least exciting suburbs of London, unless you happen to be a football enthusiast. The food at the Paris meeting was superior to that provided at Wembley but perhaps this only reflects the difference in national attitudes to eating. In Paris people happily paid about £4 for their lunch tickets whereas at Wembley many complained at being asked to pay £1.50. The difference in price is probably a fair reflection of the relative merits of the food supplied.

To summarise: this symposium was a meeting at which the opportunity to take a leisurely look at current Technicon instrumentation, to talk at length with Technicon staff, to renew acquaintances with fellow "autoanalysts", and to make new acquaintances probably compensated for the rather unexciting standard of the papers presented.

*Derrick G Porter*

### Continuous dust monitoring

A joint meeting on the above topic, organized by the Automatic Methods Group of the Chemical Society, UK, Analytical Division and the Industrial Analytical Instrumentation Group of the Institute of Measurement and Control, was held in London on 13 December 1979.

In the morning session Dr Leck and Dr Blackford [1] presented an account of the design, development and field trials of an instrument for the continuous monitoring of respirable airborne dust in coal mines. This portable, battery operated instrument (SIMSLIN-Safety In Mines Scattered Light Instrument) provides digital displays of instantaneous and average dust levels and incorporates a novel memory device that can be read back to provide an analogue trace of dust levels over a 12hr period. The second paper, presented by Mr Wallin [2], discussed a number of systems for monitoring emissions from stacks and ducts. Standard extractive systems were reviewed, followed by a description of a number of on-line (real-time) monitoring systems involving the extraction of representative stack samples. Finally, the problems associated with *in-situ* monitoring of dust levels or chemical compositions of dusts were discussed. The final paper of the morning session, given by Mr Ross [3], described a relatively simple and inexpensive optical dust monitor which had been developed to monitor powder emissions from a detergent factory. The instrument responds to changes in the magnitude of a low-frequency reflection signal resulting from turbulence in the stack/duct air-flow. Its operation, advantages and disadvantages were discussed.

The Automatic Methods Group held a short AGM before the meeting reconvened after lunch to hear the first presentation of the afternoon session in which Dr Prater [4] described the use of Directional Dust Gauges to monitor air pollution in and around a steel works. The gauge, which is cheap and uses no power, was described and the effects of wind direction and speed, gauge orientation and height above ground level on the data obtained were discussed. Mr Snowsill [5] described two devices which had been developed to monitor emissions from power stations; one used the attenuation of a light beam to give a measure of the dust in a stack emission, the other worked on an optical back-scattering system. The advantages and disadvantages of these devices were discussed and much useful information given on ways of eliminating contamination and dirtying of the optical system. Finally,, Mr Lillenfeld [6] described a number of monitoring instruments developed by his company. He paid particular attention to a silicon photodetector optical instrument which automatically monitored light scattered from dust (viz. SIMSLIN, described by the first speakers) and a fibrous aerosol monitor for asbestos monitoring which used a combination of electrical alignment of the fibres, followed by optical detection. The calibration and performance of this monitor was compared to the standard NIOSH optical-counting filter method.

The meeting was very interesting, with a wide cross-section of ideas being presented, and was much appreciated by an audience of just under 100; an attendance which was gratifying to the organisers and speakers alike.

REFERENCES

[1] 'The Design and Field Use of SIMSLIN-II - a Continuously Recording Dust Sampling Instrument' by Dr M J Leck and Dr D B Blackford (Research and Laboratory Services Division, Health and Safety Executive, Sheffield).

[2] 'Source Monitoring Systems for Particulates' by Mr S C Wallin (Warren Spring Laboratory, Stevenage).

[3] 'Monitor for Low Level Dust Emissions' by Mr R I Ross (Unilever Ltd., Engineering Division, London).

[4] 'The Application of Directional Dust Gauges to Airborne Dust Monitoring at British Steel's Redcar Development' by Dr B E Prater (British Steel Corporation, Teeside Laboratories).

[5] CEGB, Experience of On-stream Dust Monitoring' by Mr W L Snowsill (Central Electricity Research Laboratories, Leatherhead).

[6] 'Asbestos Monitoring Instrumentation' by Mr P Lillenfeld (G.C.A. Technology, Massachusetts, USA).

*Clive J. Jackson*

### New Literature

### Liquid chromatography application

'The determination of herbicide photolysis products by LC/MS' by R. F. Skinner, Q. Thomas, J. Giles and D. G. Crosby, is a recent publication from the Finnigan Corporation. Oryzalin, a pre-emergency herbicide, and its degradation products have been analyzed by Liquid chromatography/mass spectrometry (LC/MS). This study illustrates the use of Finnigan's belt type interface in LC/MS analysis.

*Copies of the report are available from Finnigan Corporation, 845 West Maude Ave, Sunnyvale, California 94086, USA.*

### Densitometer

The SD 3000 spectrodensitometer from Schoeffel Instruments for the quantitative analysis of thin layer chromatograms is described in an 8-page brochure published by the Schoeffel Instrument Division of Kratos. The SD 3000 is a thin layer chromatogram scanner capable of making absorbance and fluorescence measurements of spotted thin layer separations on TLC plates, paper or film as well as agarose gels, disc gels, slab gels and electrophoretic media. The densitometer is modular in design. Accessories include the necessary carriers for various media, and an electronic reporting integrator for thorough quantitative analysis reports.

*The brochure is available from Kratos Inc, Schoeffel Instrument Division, 24 Booker St, Westwood, NJ 07675, USA.*

### Spectroscopic study of liquids and gases

This report, 'A CARS system for the study of liquids and gases' by D. R. Williams and I A. Stenhouse details the technique of coherent anti-strokes raman spectroscopy (CARS). The basic theory of the technique is given and an experimental CARS system for the study of liquids and gases is described. The system was commissioned by observing the CARS spectra of liquids.

*Copies of the report, price £1.00, are available from HMSO, 49 High Holborn, London WC1V 6HB, UK.*

### Liquid chromatography

High performance liquid chromatography and its application in pharmaceutical analytical problems is the topic of a new book now available from Hewlett Packard. The 157-page book reviews the use of HPLC for pharmaceutical measurements in terms of applications and methodology. Prime applications such as isolation of natural compounds, pharmacokinetics and drug metabolism are described with sample chromatograms and illustrative figures. Sections on methodology explain sample preparation procedures, separation procedures, separation techniques, detectors and quantitative analysis.

*'High performance liquid chromatography in pharmacuetical analyses' is available free of charge from Hewlett-Packard Ltd, King Street Lane, Winnersh, Wokingham, Berks, UK.*

### Automatic data acquisition

'Automatic data acquisition and processing with the APEX goniometer, PDP 11/03 and IBM 370 computer, with application to surface texture studies of magnox fuel cladding', by M.O. Boles, B.A. Bellamy and G.A. Burras, is the title of a recent publication from HMSO. It takes the form of a working manual enabling the user to operate the described system and make modifications to suit individual requirements. Part one describes the general procedures required for automatic data acquisition and processing. Part two describes in detail the application of automatic data collection to texture studies of magnox fuel cladding.

*Copies of the report are available from HMSO, PO Box 569, London SE1 9NH, price £2.00.*

### Phototitration book

A 160 page book on methods of analysis using photometric end-point sensing has been published by Mettler which covers the theory of titration. Detailed methods are suggested for many metals, including calcium, magnesium, aluminium, etc and for dissolved oxygen, sulphate, fluorine, halogens and vitamin C. The book contains an extensive section giving literature references for analytical methods using phototitration.

*'Phototitration - a guide to methods' by K Mooibroek is available from MSE Scientific Instruments, Manor Royal, Crawley, West Sussex, UK, price £25.00.*

